# Retrospective analysis of effectiveness of fingolimod in real life setting in Turkey (REFINE)

**DOI:** 10.55730/1300-0144.5588

**Published:** 2022-11-30

**Authors:** Aslı TUNCER, Murat KÜRTÜNCÜ, Murat TERZİ, Uğur UYGUNOĞLU, Cansu GÖNCÜOĞLU, Ayşe Nur YÜCEYAR, Özgül EKMEKÇİ, Recai TÜRKOĞLU, Aysun SOYSAL, Mesrure KÖSEOĞLU, Cavit BOZ, Yeşim BECKMANN, Ömer Faruk TURAN, Meltem DEMİRKIRAN, Gülşen AKMAN, Burcu ALTUNRENDE, İlknur AYDIN CANTÜRK, Erkingül BİRDAY, Abdulcemal ÖZCAN, Özden KAMIŞLI, Nazire Pınar ACAR ÖZEN, Rabia Gökçen GÖZÜBATIK ÇELİK, Belgin PETEK BALCI, Hüsnü EFENDİ, Cansu SARIKAYA, Aylin AKÇALI, Münire KILINÇ, Sibel CANBAZ KABAY, Ferah KIZILAY, Serhan SEVİM, Gülcan BARAN GAZALOĞLU, Caner Feyzi DEMİR, Ferhat BALGETİR, Nefati KIYLIOĞLU, Hande SARIAHMETOĞLU, Çağcan ÖLMEZ, Kamil MAVİ, Süha YÜKSEL, Nihal IŞIK, Sabahattin SAİP, Rana KARABUDAK, Aksel SİVA, Mefküre ERAKSOY

**Affiliations:** 1 Department of Neurology, Faculty of Medicine, Hacettepe University, Ankara, Turkey; 2 Department of Neurology, İstanbul Faculty of Medicine (ÇAPA), İstanbul University, İstanbul, Turkey; 3 Department of Neurology, Faculty of Medicine, Samsun Ondokuz Mayıs University, Samsun, Turkey; 4 Department of Neurology, Faculty of Medicine, İstanbul University Cerrahpaşa, İstanbul, Turkey; 5 Department of Clinical Pharmacy, Faculty of Pharmacy, Hacettepe University, Ankara, Turkey; 6 Department of Neurology, Faculty of Medicine, Ege University, İzmir, Turkey; 7 İstanbul Haydarpasa Numune Training and Research Hospital, University of Health Sciences, İstanbul, Turkey; 8 Bakırköy Psychiatric and Neurological Diseases Hospital, İstanbul, Turkey; 9 Department of Neurology, Faculty of Medicine, Karadeniz Technical University, Trabzon, Turkey; 10 Department of Neurology, Faculty of Medicine, İzmir Kâtip Çelebi University, İzmir, Turkey; 11 Department of Neurology, Faculty of Medicine, Uludağ University, Bursa, Turkey; 12 Department of Neurology, Faculty of Medicine, Çukurova University, Adana, Turkey; 13 Florence Nightingale Hospital, Science University, İstanbul, Turkey; 14 Göztepe Professor Doctor Süleyman Yalçın City Hospital, İstanbul, Turkey; 15 Department of Neurology, Faculty of Medicine, Medipol University, İstanbul, Turkey; 16 Department of Neurology, Faculty of Medicine, İnönü University, Malatya, Turkey; 17 Haseki Training and Research Hospital, İstanbul, Turkey; 18 Department of Neurology, Faculty of Medicine, Kocaeli University, Kocaeli, Turkey; 19 Department of Neurology, Faculty of Medicine, Gaziantep University, Gaziantep, Turkey; 20 Başkent University Hospital, Ankara, Turkey; 21 Department of Neurology, Faculty of Medicine, Kütahya Health Sciences University, Kütahya, Turkey; 22 Department of Neurology, Faculty of Medicine, Akdeniz University, Antalya, Turkey; 23 Department of Neurology, Faculty of Medicine, Mersin University, Mersin, Turkey; 24 Department of Neurology, Faculty of Medicine, Fırat University, Elazığ, Turkey; 25 Department of Neurology, Faculty of Medicine, Adnan Menderes University, Aydın, Turkey; 26 Novartis Health Food and Agriculture Products Industry and Trade Inc., İstanbul, Turkey; 27 Okan University Hospital, İstanbul, Turkey

**Keywords:** Fingolimod, disease modifying treatment, treatment switch, glatiramer acetate, beta interferon

## Abstract

**Background/aim:**

During multiple sclerosis (MS) treatment different modes of action such as lateral (interferon beta to glatiramer acetate or glatiramer acetate to interferon beta) or vertical (interferon beta/glatiramer acetate to fingolimod) drug switch can be performed. This study aims to investigate the clinical effectiveness of switching from the first-line injectable disease modifying treatments (iDMTs) to fingolimod (FNG) compared to switching between first-line iDMTs.

**Materials and methods:**

This is a multicenter, observational and retrospective study of patients with relapsing-remitting MS who had lateral and vertical switch. The observation period included three key assessment time points (before the switch, at switch, and after the switch). Data were collected from the MS patients’ database by neurologists between January 2018 and June 2019. The longest follow-up period of the patients was determined as 24 months after the switch.

**Results:**

In 462 MS patients that were included in the study, both treatments significantly decreased the number of relapses during the postswitch 12 months versus preswitch one year while patients in the FNG group experienced significantly fewer relapses compared to iDMT cohort in the postswitch 12 months period. FNG cohort experienced fewer relapses than in the iDMT cohort within the postswitch 2 year. The mean time to first relapse after the switch was significantly longer in the FNG group.

**Conclusion:**

The present study revealed superior effectiveness of vertical switch over lateral switch regarding the improvement in relapse outcomes. Patients in the FNG cohort experienced sustainably fewer relapses during the follow-up period after the switch compared the iDMT cohort. Importantly, switching to FNG was more effective in delaying time to first relapse when compared with iDMTs.

## 1. Introduction

Around 85%–90% of multiple sclerosis (MS) patients initially present with the relapsing-remitting form of the disease (RRMS) [1–3]. Treating persons with RRMS (pwRRMS) timely and effectively is crucial since transition to secondary progressive MS occurs in approximately 50% of patients over 15 to 20 years after the onset of the disease [4–6].

Disease-modifying therapies (DMTs) constitute the mainstay of the treatment. By the European Committee of Treatment and Research in Multiple Sclerosis (ECTRIMS) and the European Academy of Neurology (EAN) 2018, the early initiation of a DMT is recommended for all MS patients in order to prevent possible neurological disability progression in the later phases [1]. Interferon-beta (IFN β 1a and 1b) and glatiramer acetate (GA) are the two injectable DMTs (iDMT) that have been used for the treatment of MS since the 1990s [7]. Switching to a more efficacious drug is recommended in patients with active disease. Not only patient characteristics and comorbidities but also drug safety profile and disease severity should be considered while making a treatment decision [1, 8]. Several compounds with different modes of action have become available for the treatment of pwRRMS [1, 8, 9].

Fingolimod (FNG), a sphingosine 1-phosphate receptor (S1PR) modulator is one of those treatment options. It is the first oral DMT approved for the treatment of MS [10]. It is indicated in the treatment of pwRRMS [10, 11].The efficacy of FNG in slowing down the disease activity and disability progression was established in the randomized controlled trials as FREEDOMS, FREEDOMS 2, and TRANSFORMS trials [12–14]. It was also confirmed by several real-life studies from various geographic regions [15–20]. However, to the best of our knowledge, the comparative effectiveness of FNG with the other iDMTs in real life has been evaluated in a few studies so far [21–25]. In this context, gathering further clinical information on this issue may surely help clinicians while making treatment decisions in daily practice.

This study aimed to determine the clinical effectiveness of switching from f﻿irst-line iDMTs to FNG in comparison with switching between first-line iDMTs.

## 2. Materials and methods

### 2.1. Study design and setting

This multicenter, observational study was based on a retrospective review of data from pwRRMS who switched from an iDMT (interferon beta or glatiramer acetate) to another one (iDMT cohort) or fingolimod (FNG cohort). The observation period was at least two years and included three key assessment time points: T1 (12 months before the switch), T2 (at the switch), T3 (12 months after the switch). Further follow-up (24 months after the switch) was possible for a group of patients. Data were retrieved between January 2018 and June 2019 from databases that collect observational data about MS patients as part of routine clinical care or hospital medical records.

Since only neurologists practicing at tertiary hospitals are authorized to initiate and switch DMTs according to the local reimbursement regulations, the data was collected from the neurology clinics of 24 tertiary health care institutions (the University or the Training and Research Hospitals) across Turkey. Therefore, the collected data is originated from highly specialized MS centers and may be representative of pwRRMS in Turkey.

Switching to high-efficacy DMTs when treatment is initiated with moderate efficacy DMTs and encountered with breakthrough disease activity is called vertical switch, the change between treatments with similar effectiveness is called lateral switch. The data of annualized relapse rate (ARR), the expanded disability status scale (EDSS), radiological activity, treatment decisions, tolerability of drugs, progression of the disease as well as patient demographics and MS and treatment history were reviewed. Annualized relapse rate was calculated by dividing the number of relapses in the obtained data by the observation period (year). Progression index (PI) was calculated by EDSS scores divided by years since their clinical diagnosis. Lesion load indicates the total number of lesions detected on magnetic resonance imaging.

### 2.2. Study population

The inclusion criteria for this study were as follows: pwRRMS ≥18 years of age on the date of treatment change, diagnosis of MS for 2 to 15 years before the switching date (fulfillment of the revised McDonald criteria [26]); treatment with a first-line iDMT during at least 12 months before treatment change, switch from a first-line iDMT to another one (lateral switch) or to fingolimod (vertical switch) between February 2015 and September 2016 (extended from January 2000 to September 2016 for iDMT cohort via a protocol amendment to ensure inclusion of a preplanned number of patients),≥1 relapse during the 12 months before the treatment change, (therefore pwRRMS who required a treatment switch because of an inadequate efficacy), postswitch follow-up of at least 12 months (cut-off date for follow-up was 30 September 2017), complete medical history throughout the observation period, followed up by the participating study center at study inclusion.

Exclusion criteria were based on the patients’ status on the date of treatment change. Patients with primary or secondary progressive MS, patients who received natalizumab, and patients with a significant comorbid systemic disease that could affect the management of MS were excluded from the study.

### 2.3. Statistical analysis

Independent variables such as the proportion of patients without relapses, the ARR and relapses requiring corticosteroids, the mean EDSS scores, the mean number of lesions on T2-weighted scan and Gadolinium (Gd) enhancing lesions on T1-weighted scan, the time to first relapse (month) after the treatment change and patient demographics (age, sex, disease duration, number of relapses in the 12 months prior and after switch, prior MS treatments) were analyzed with the IBM Statistical Package for Social Science v.23 (Armonk, NY, USA) software. Quantitative variables were summarized as mean, standard deviation, median, maximum and minimum values. Qualitative variables were summarized as frequency, and percentages. While calculating the group time interaction of the parameters measured at 3 different time points, repeated measure ANOVA analysis was used.

Visual (histograms, probability plots) and analytical methods (Kolmogorov-Smirnov/Shapiro-Wilk tests) were used to determine the distribution of variables. In the comparison of the iDMT and FNG cohorts, the t-test was used for normally distributed continuous variables, and the Mann-Whitney U test was used for nonnormally distributed continuous variables. In the comparison of the categorical variables chi-square test was used. Survival analysis was performed by Kaplan-Meier method. The graphical representations were generated by various R statistical packages. p < 0.05 was considered statistically significant.

### 2.4. Ethics

The study was approved by Hacettepe University Clinical Trials Ethics Committee in line with local regulations.

## 3. Results

We included 462 pwRRMS (73.8% females) whose treatments were switched to iDMTor FNG. Five patients were excluded due to irregular follow-up and finally, the groups constituted 224 patients for iDMT and 233 patients for the FNG cohort (Table 1).

Although the 2 cohorts showed basically comparable parameters, the mean age of the iDMT cohort was higher than the FNG cohort (41.2 ± 9.0 vs. 38.8 ± 8.2, mean ± SD, p = 0.003). Both groups were comparable regarding other demographic feature such as sex, education, and occupation. In both groups, the majority of the patients were unemployed and the age at MS diagnosis was around midtwenties. The age at diagnosis and onset of disease was similar in both groups (Table 1). The median time between the onset of symptoms and diagnosis was 5.6 months in the iDMT cohort (min-max: 0–192.8; IQR: 25.9 months) and 4.2 months in the FNG cohort (min-max: 0–187.1; IQR: 23.9) (p = 0.639). The initial presentation was monosymptomatic in most of the patients (91.4% in iDMT and 87.7% in the FNG cohort) and did not differ between the groups (p = 0.202).

The reason for the switch was comparable in both study groups. Ineffectiveness was the cause of treatment change in more than 90% of patients. The duration from diagnosis to treatment change was shorter in the iDMT cohort compared with the FNG cohort (53.1 ± 37.2 vs. 81.8 ± 52.7,p < 0.001) (Table 1).

The mean duration between the first two relapses following the onset of symptoms was comparable in the study groups (25.1 ± 2.1 months in the iDMT vs. 25.1 ± 1.9 months in the FNG cohorts, p = 0.997). During the preswitch 12 months, the mean ARR in the cohorts were similar (FNG: 1.3 ± 0.04 vs. iDMT: 1.4 ± 0.06, p = 0.266). However, in the same time period the mean number of corticosteroid-requiring relapses in the iDMT cohort was higher than the FNG cohort (1.4 ± 0.9 vs. 1.2 ± 0.6, p = 0.004). Both treatments significantly decreased the number of relapses during the post switch 12 months versus preswitch one year. However, patients in the FNG group had significantly fewer ARR compared to the iDMT cohort in the postswitch 12 months period (0.3 ± 0.04 vs. 0.5 ± 0.05, p < 0.001). Nevertheless, there was no difference in the number of steroid-requiring relapses (iDMT cohort: 1.2 ± 0.9 vs. FNG cohort: 1.2 ± 0.7, p = 0.861).

**Figure 1:**
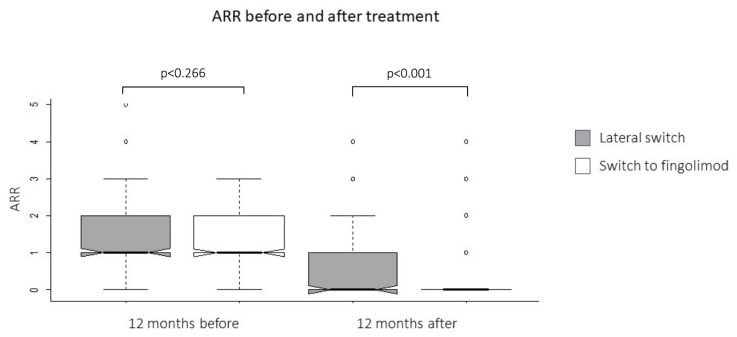
Annualized relapse rates in the one year before and after the switch. The middle lines of the notched box plots refer to the medians. Note the large difference in the ARR between the groups in the first year after the switch despite the comparable ARR at baseline (p < 0.001, Cohen’s d = –0.6).

**Figure 2:**
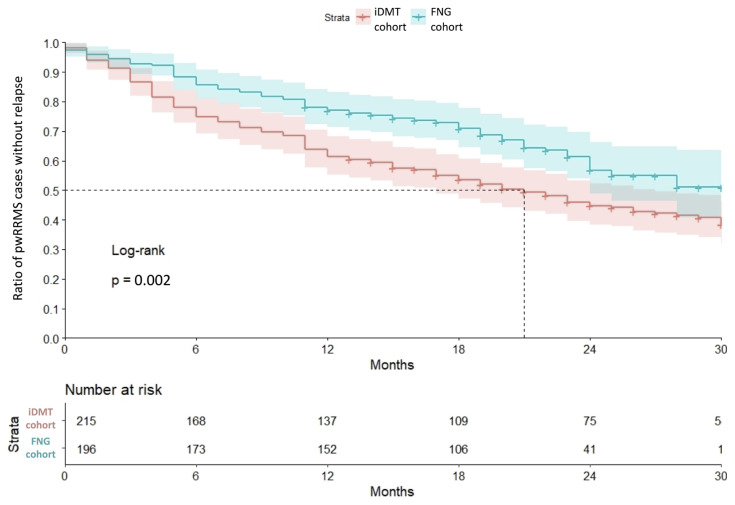
Time to first relapse after the switch.

FNG cohort experienced fewer relapses (0.2 ± 0.04 vs. 0.9 ± 0.09) than in the iDMT cohort within the further follow-up period (p < 0.001) (Figure 1). The FNG cohort had less steroid requiring relapses compared with the iDMT cohort (1.4 ± 0.7 vs. 1.1 ± 0.4; p = 0.027) within the further follow-up period. The mean time to first relapse after the switch was significantly longer in the FNG group, log-rank test: p = 0.002) (Figure 2).

**Figure 3:**
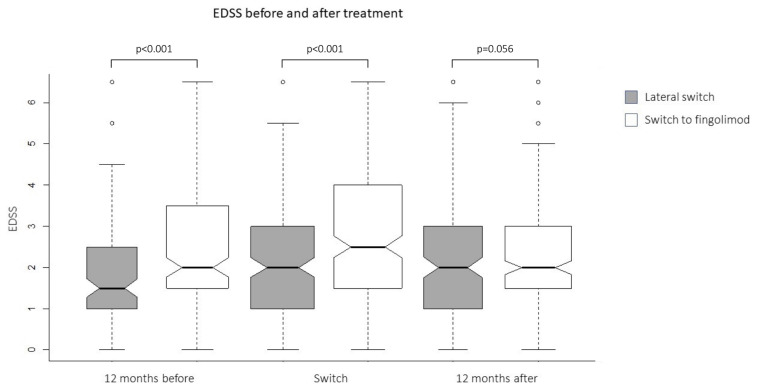
EDSS 12 months before the switch, at the time of the switch, and 12 months after the switch. There was no difference in terms of time-dependent EDSS change between the groups. The middle lines of the notched box plots refer to the medians. There was no difference in the EDSS between the treatment arms after 12 months despite the patients in the fingolimod group had higher EDSS 12 months before and at the time of switch.

The mean EDSS score 12 months before and at the treatment change were higher in the FNG cohort (2.4 ± 0.10 vs. 1.8 ± 0.12, p < 0.001 and 2.6 ± 0.10 vs. 2.1 ± 0.10, p < 0.001, respectively). No significant difference was found between FNG and iDMT cohorts, regarding the postswitch 12 months EDSS (2.3 ± 0.10, 2.0 ± 0.12, p = 0.056) (Figure 3).

**Figure 4:**
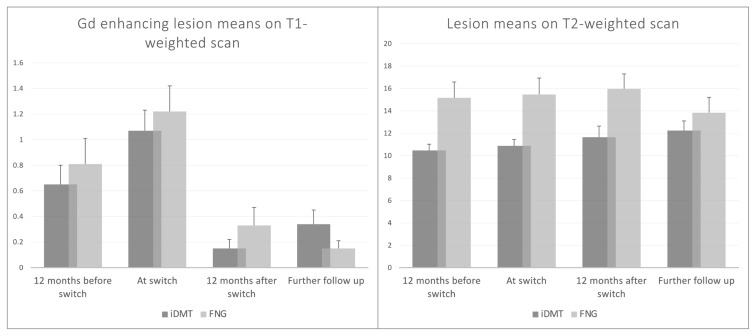
Changes in the mean number of T2 lesions on T2 weighted scan (a) and Gd enhancing lesions on T1 weighted scan (b) on MRI scans.

A summary of the MRI findings is presented in Table 2. The mean number of Gd-enhancing lesions at four consecutive time points (12 months before switch, at the time of the switch, postswitch 12 months, and further follow-up) did not differ between the groups. However, the groups differed regarding the mean number of T2 lesions at all time points except the further follow-up (Table 2, Figures 4a and 4b).

Regarding the first dose administration data, symptomatic bradycardia had occurred in 7 (3.0%) patients whereas prolonged monitorization was required only in 5 patients (2.2%).

## 4. Discussion

In the REFINE study, we retrospectively compared the effects of vertical (IFN/GA to FNG) and lateral (IFN to GA or GA to IFN) treatment changes in 457 pwRRMS with a follow-up of at least 12 months.

Consistent with the studies from various geographic regions around the world [15–25, 27], more than two thirds of patients were females in the present study. We noticed that, although treatment naïve patients were not included in the REFINE study, the overall patient population was younger than in many other previously reported studies. Several studies from populations of Middle East reported mean patient ages much closer to what we observed in the REFINE study [19, 27]. The study findings pointed out a tendency towards switching from IM to SC iDMTs before escalation to FNG. This was in line with the longer period of time between diagnosis and switch in the FNG cohort than in the iDMT cohort. A similar switch pattern was reported in an international retrospective study analyzing MSBase registry data sets from various geographic regions all around the world [22]. The majority of patients who used GA as previous therapy switched to FNG not the other first line iDMT. We do not have the data of all switch behavior of neurologists in our country. In this cohort, it may origin from the traditional behavior of neurologists to choose second line therapy after GA.

The study showed superior effectiveness of vertical switch over lateral switch regarding the improvement in relapse outcomes. Patients in the FNG cohort experienced sustainably fewer relapses during the further follow-up after the switch compared with the patients in the iDMT cohort. Additionally, switching to FNG was more effective in delaying time to first relapse compared with iDMTs.

The comparative effectiveness of FNG versus iDMTs has been assessed in three interventional randomized controlled [14, 25, 28] and 5 retrospective observational studies [21–25]. Phase 3 TRANSFORMS study, an initial study comparing FNG with IFN beta 1-aIM demonstrated that, compared with IFN beta 1-a, FNG was more efficacious in improving the ARR and delaying the time to first relapse. However, there was no difference in the disability progression [14]. Three of these retrospective studies were based on the analysis of United States claims databases whereas the other 2 studies retrieved data from the MSBase registry internationally and NeuroTransData (NTD) network in Germany, respectively [21–25]. In these studies, FNG significantly improved the ARR and was superior to its comparator iDMTs regarding the improvement in ARRs [21–25] and delaying the time to first relapse [21, 22, 24]. However, the comparative effectiveness on ARR improvement versus iDMTs varied markedly across the studies as indicated by 26% to 62% reductions in relapses per year [21–25]. The difference in efficacy between the switching to IFN beta 1a 44 mcg or FNG was studied in a retrospective study involving 92 patients. Although follow-up periods of this study and ours are similar, our study is more advantageous in terms of the sample size [29]. In the study of D’amico et al., lateral switch to IFN beta 1a 44 mcg and escalation switch to FNG showed no difference for disease activity at 24 months [29].

Overall, our findings on relapse outcomes were consistent with the previous randomized controlled trials. It is worth mentioning that the patients in our study were followed for a longer period (up to 30 months) compared with these studies and the superiority regarding the proportion of patients with no relapse remained sustainably and significantly higher with FNG compared with iDMTs. Additionally, contrasting with the TRANSFORMS study, the patients in the FNG arm in our study were more disabled than the ones in the iDMT cohort at baseline. Despite worse EDSS at switch time, we observed an improvement in EDSS only in patients treated with FNG at the postswitch 12 month, but it did not reveal a statistically significance. Also, there was no difference between the groups in term of PI scores. The impact of FNG on disability progression versus iDMTs in real life was previously assessed in two clinical database studies [21, 22]. Although the assessment parameters in those studies (proportion of patients without confirmed EDSS progression at month 3) differed from the one in the REFINE study (mean EDSS), all 3 studies were consistent regarding the beneficial effects of FNG on disability progression over iDMTs.

Recently, the ASSESS study investigated the efficacy of both 0.5 mg and 0.25 mg once daily doses of FNGvs.20 mg daily GA. This study demonstrated that FNG 0.5 mg/day was superior to GA in reducing ARR, delaying the time to first relapse, increasing the proportion of patients with no relapse. Furthermore, compared with GA, FNG 0.5 mg reduced the development of Gd enhancing and new or enlarging T2 lesions by 56% and 54%, respectively [30]. The 18-month, multicenter, randomized, open-label GOLDEN study also confirmed the beneficial effects of FNG over IFN beta 1-a regarding clinical and MRI outcomes. Sixty-one percent of relative reduction in ARR and a significant reduction in MRI lesions over 18 months were demonstrated in favor of FNG group [31]. Regarding MRI findings, we did not observe an improvement in Gd enhancing lesions versus iDMTs in contrast to the TRANSFORMS and GOLDENS studies. Although not statistically significant, we observed a reduction in the number of Gd enhancing on T1weighted and T2 weighted scan lesion load in favor of FNG over time. Besides the FNG cohort displayed higher disease burden as evidenced by lesion load at switch compared to the iDMT group. This observation is valuable since, to our knowledge, FNG and iDMTs have not been compared in a parallel-group real life study regarding their impacts on MRI outcomes. However, this outcome needs to be interpreted cautiously and confirmed in future studies.

At this point, it is noteworthy to mention that several points should be taken into consideration while evaluating our findings. This multicenter, observational and retrospective study may lead to reporting bias while collection of the data. The patient populations in the real life studies, as expected, were heterogenous regarding study characteristics (design, duration, comparator, length, eligibility criteria) as well as several demographic and disease characteristics. This is probably the main reason for the numerical variations across the studies. In two previous real-life studies, it was reported that age alone did not have an impact on the comparative effectiveness of FNG and DMTs on ARR [23, 25]. It nevertheless deserves emphasizing that, in the REFINE study, the imbalances regarding disease characteristics were all in favor of iDMTs except steroid requiring relapses in the year before the switch. Therefore, the differences in clinical and demographic characteristics might have had a relatively limited impact on the superior effectiveness of FNG over iDMTs in present study.

Despite the limitations of retrospective data collection studies, the REFINE study has several strengths. First, the present study, to the best of our knowledge, is the only study that investigated the comparative effectiveness of FNG versus iDMTs regarding both clinical and radiological outcomes. Secondly, the data obtained from 24 neurology clinics from various regions of the country is representative for MS population in Turkey and at least partly reflects treatment approach of Turkish neurologists in routine clinical practice.

In conclusion, switching from iDMTs to FNG delayed the time to first relapse and, displayed a trend toward improvement in MRI lesions compared to switching between iDMTs. Overall, these results confirm the findings of previous randomized-controlled and real-life studies comparing FNG with iDMTs.

## Figures and Tables

**Table 1:** Patient characteristics.

Characteristics	iDMT cohort	FNG cohort	p
**Age, years, mean (±SD)**	41.2 (±9.0)	38.8 (±8.2)	**0.003**
**Sex, female, n (%)**	171 (76.3)	165 (70.8)	0.181
**Body mass index, kg/m** **^2^** **, mean (±SD)**	24.4 (±4.5)	24.4 (±3.8)	0.987
**Age at diagnosis of MS, years, mean (±SD)**	27.4 (±8.2)	26.6 (±7.5)	0.303
**Age at onset of symptoms, years, mean (±SD)**	25.6 (±8.1)	25.1 (±7.6)	0.480
**Duration from diagnosis to switch, months, mean (±SD)**	53.1 (±37.2)	81.8 (±52.7)	**<0.001**
**Education, n (%)**			
University	101 (53.4)	104 (47.3)	0.377
High school	37 (19.6)	54 (24.5)	
Primary/middle school	51 (27.0)	62 (28.2)	
**Occupation, n (%)**			
Public servant	22 (12.0)	21 (9.9)	0.744
Worker	31 (16.8)	40 (18.8)	
Private sector	49 (26.6)	50 (23.5)	
Housewife/unemployed	82 (44.6)	102 (47.9)	
**Presentation at onset, n (%)**			
Monosymptomatic	202 (91.4)	200 (87.7)	0.202
Polysymptomatic	19 (8.6)	28 (12.3)	
**DMT used before switch, n (%)**			
IFN beta	194 (87.0)	165 (71.4)	**<0.001**
Glatiramer acetate	29 (13.0)	66 (28.6)	
**Reason for switch, n (%)**			
Ineffectiveness	201 (90.5)	211 (90.9)	0.938
Side effect	8 (3.6)	10 (4.3)	
Ineffectiveness and side effect	7 (3.2)	6 (2.6)	
Incompliance	6 (2.7)	5 (2.2)	

iDMT: injectable disease-modifying therapy; IFN: interferon; FNG: fingolimod; SD: standard deviation, n: number of patients.

**Table 2:** EDSS, PI, ARR values and lesion load of the patients.

	iDMT cohort		FNG cohort		p
EDSS scores	n	mean (±SEM)	n	mean (±SEM)	
12 months before switch	120	1.8 (±0.12)	180	2.4 (±0.10)	**<0.001**
At switch	184	2.1 (±0.10)	218	2.6 (±0.10)	**<0.001**
12 months after switch	160	2.0 (±0.12)	206	2.3 (±0.10)	0.056
Interaction of group and time, mean (±SD)	97	2.0 (±0.14)	160	2.5 (±0.11)	0.870
**PI scores**					
12 months before switch	120	0.5 (±0.06)	180	0.5 (±0.03)	0.685
At switch	184	0.6 (±0.04)	218	0.6 (±0.04)	0.500
12 months after switch	160	0.6 (±0.05)	206	0.5 (±0.03)	0.056
Interaction of group and time, mean (±SD)	97	0.6 (±0.06)	160	0.5 (±0.04)	0.574
**ARR**					
12 months before switch	223	1.4 (±0.06)	233	1.3 (±0.04)	0.266
12 months after switch	223	0.5 (±0.05)	232	0.3 (±0.04)	**<0.001**
Further follow-up	200	0.9 (±0.09)	190	0.2 (±0.04)	**<0.001**
Interaction of group and time, mean (±SD)	198	0.9 (±0.04)	189	0.6 (±0.04)	**<0.001**
**Gd-enhancing lesion load on T1 weighted scan**					
12 months before switch	51	0.7 (±0.15)	54	0.8 (±0.20)	0.505
At switch	90	1.07 (±0.2)	90	1.2 (±0.2)	0.558
12 months after switch	66	0.2 (±0.1)	63	0.3 (±0.1)	0.256
Further follow-up	71	0.3 (±0.1)	65	0.2 (±0.1)	0.141
**Lesion load on T2 weighted scan**					
12 months before switch	57	10.5 (±0.56)	63	15.2 (±1.42)	**0.003**
At switch	115	10.9 (±0.58)	92	15.5 (±1.47)	**0.004**
12 months after switch	68	11.7 (±0.99)	72	16.0 (±1.33)	**0.010**
Further follow-up	72	12.3 (±0.85)	67	13.8 (±1.37)	0.326

iDMT: injectable disease-modifying therapy; FNG: fingolimod; SD: standard deviation; PI: progression index; EDSS: expanded disability status scale; ARR: annualized relapse rate; n: number of patients; SEM: standard error of the mean.

Further follow-up data for EDSS and PI could not obtained.
